# Supplementary material for: Structure and assembly of double-headed Sendai virus nucleocapsids
Source: Commun Biol. 2021 Apr 22;4:494. doi: 10.1038/s42003-021-02027-y (PMC8062630; doi:10.1038/s42003-021-02027-y)
Supplement: Supplementary file 3 — Supplementary Information [file 42003_2021_2027_MOESM3_ESM.pdf]

Supplementary information for

Structure and assembly of double-headed Sendai virus nucleocapsids

Na Zhang[1,2,3,9], Hong Shan[1,9], Mingdong Liu[1,3,9], Tianhao Li[1,3,9], Rui Luo[4], Liuyan Yang[5,6], Lei Qi[2], Xiaofeng Chu[1,3], Xin Su[1,3], Rui Wang[1], Yunhui Liu[1], Wenzhi Sun[7,8], Qing-Tao Shen[1,2]✉

Affiliations:

[1]iHuman Institute, School of Life Science and Technology, ShanghaiTech University, Shanghai 201210, China.

[2]Laboratory for Marine Biology and Biotechnology, Qingdao National Laboratory for Marine Science and Technology, Qingdao 266237, China.

[3]University of Chinese Academy of Sciences, Beijing 100049, China.

[4]State Key Laboratory of Agricultural Microbiology, College of Veterinary Medicine, Huazhong Agricultural University, Wuhan 430070, China.

[5]State Key Laboratory of Microbial Technology, Marine Biotechnology Research Center, Shandong University, Qingdao 266237, China.

[6]College of Marine Life Sciences, Ocean University of China, Qingdao 266003, China.

[7]Chinese Institute for Brain Research, Beijing 102206, China.

[8]School of Basic Medical Sciences, Capital Medical University, Beijing 100069, China.

[9]These authors contributed equally: Na Zhang, Hong Shan, Mingdong Liu, Tianhao Li

✉Correspondence to: Qing-Tao Shen (shenqt@shanghaitech.edu.cn)

The PDF file includes:

Supplementary Figures 1 to 14

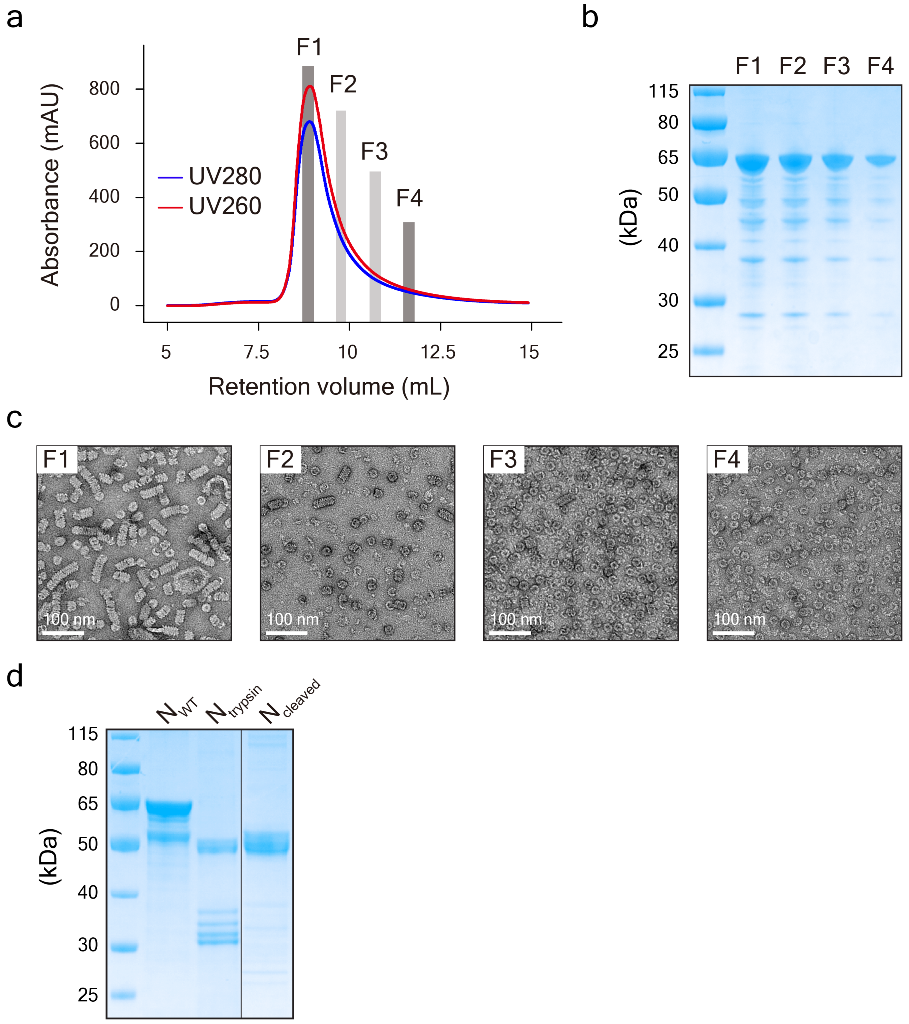

Supplementary Fig. 1 Purification of SeV nucleoprotein and its structural diversity. a Gel filtration chromatography of SeV nucleoprotein. The absorbance of UV260 and UV280 were marked in red and blue. Four fractions (F1, F2, F3 and F4, 0.2 mL per each) were selected to sample the whole peak. F1 and F4, colored in dark grey, are chosen for helical stem and clam-shaped structure analysis, respectively. **b** SDS-PAGE gel of fractions F1-F4. **c** Structural diversity of fractions F1-F4 in Negative stain EM. Comparing to F3 and F4, F1 and F2 were diluted 12 and 7 times for negative stain EM grids preparation, respectively. **d** Cleavage comparison on nucleoproteins after trypsin digestion or under storage under 4 °C for five weeks.

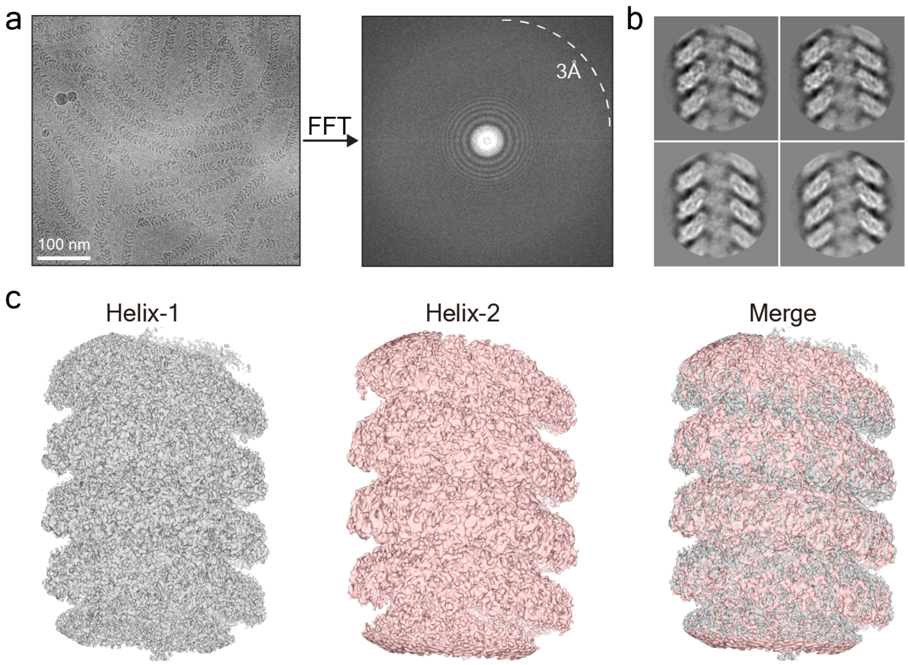

Supplementary Fig. 2 3D reconstruction of helical stems of SeV NC_{WT}. **a** A typical cryo-EM micrograph of SeV NC_{WT}, and its power spectrum. **b** 2D class average galleries of helical stems of SeV NC_{WT}. **c** 3D classification analysis on helical stems of SeV NC_{WT}. Two helical reconstructs and their merged structure are shown here.

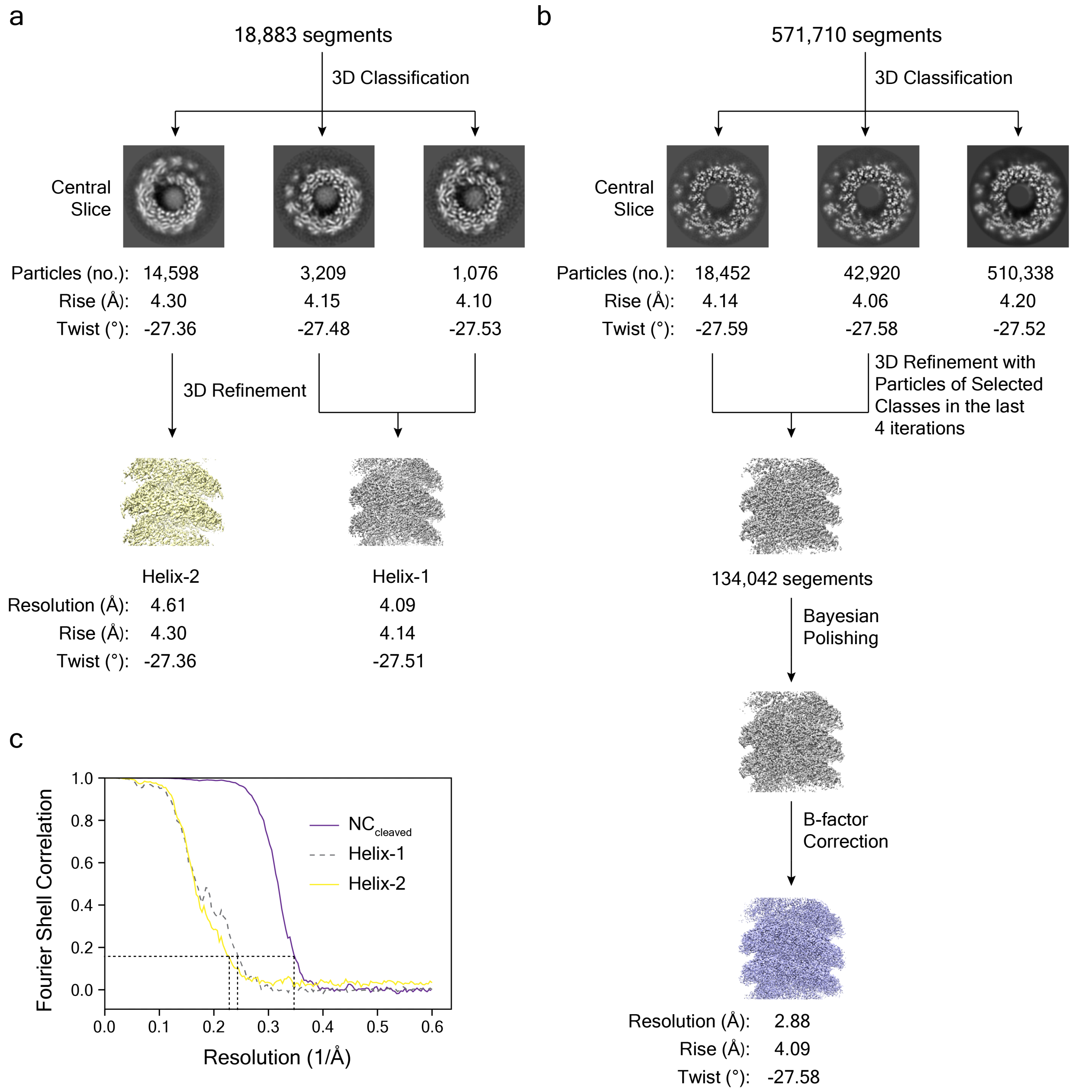

Supplementary Fig. 3 A flow chart for 3D helical reconstruction of SeV NC_{WT} and $NC_{cleaved}$, and the respective FSC curves. a A flow chart for 3D helical reconstruction on SeV NC_{WT}. **b** A flow chart for 3D helical reconstruction on SeV $NC_{cleaved}$. **c** FSC curves for SeV NC_{WT} and $NC_{cleaved}$.

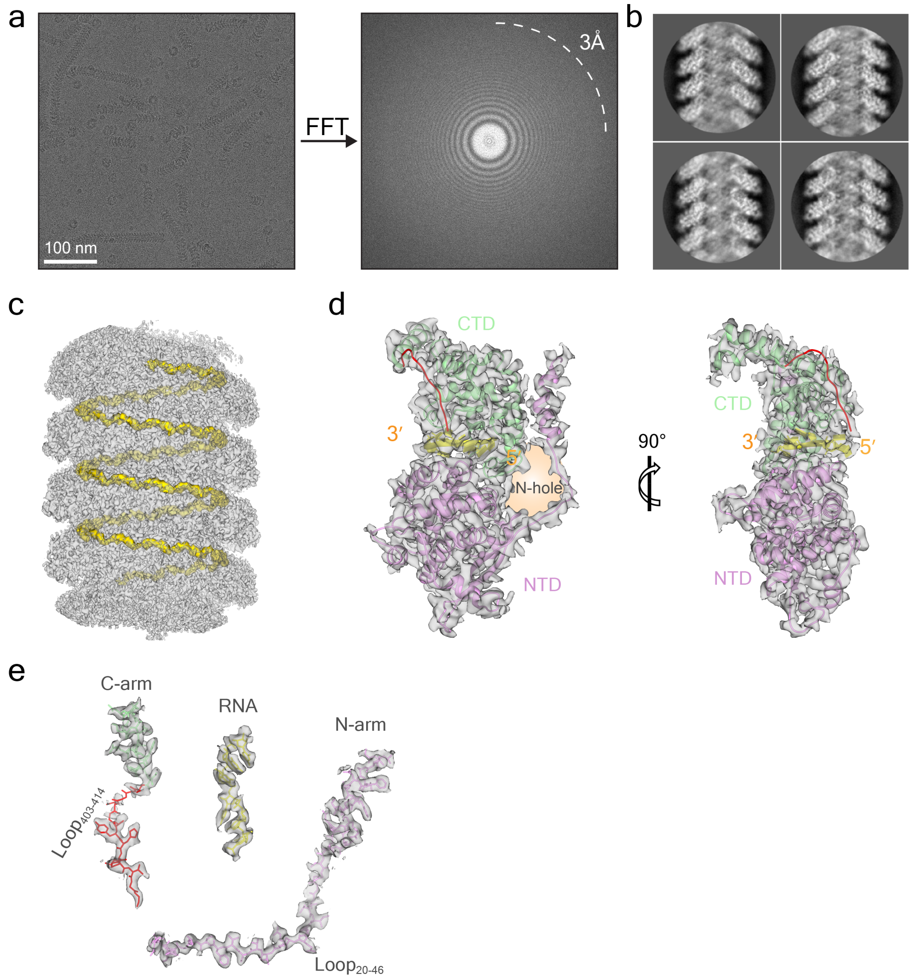

Supplementary Fig. 4 3D reconstruction of SeV NC$_{cleaved}$ helical stem. a A typical cryo-EM image of SeV NC$_{cleaved}$, and its power spectrum. **b** 2D class average galleries of helical stem of SeV NC$_{cleaved}$. **c** 3D reconstruction of helical stems of SeV NC$_{cleaved}$. RNA is colored in gold. **d** EM density of one protomer of SeV NC$_{cleaved}$ and the docked atomic model. N-hole formed by Loop$_{20\text{-}46}$, Loop$_{92\text{-}102}$ and Loop$_{312\text{-}320}$ is depicted in light orange. **e** Cryo-EM densities of selected areas of NC$_{cleaved}$ at 2.9 Å resolution. N-arm, Loop$_{20\text{-}46}$, C-arm, Loop$_{403\text{-}414}$ and RNA are shown.

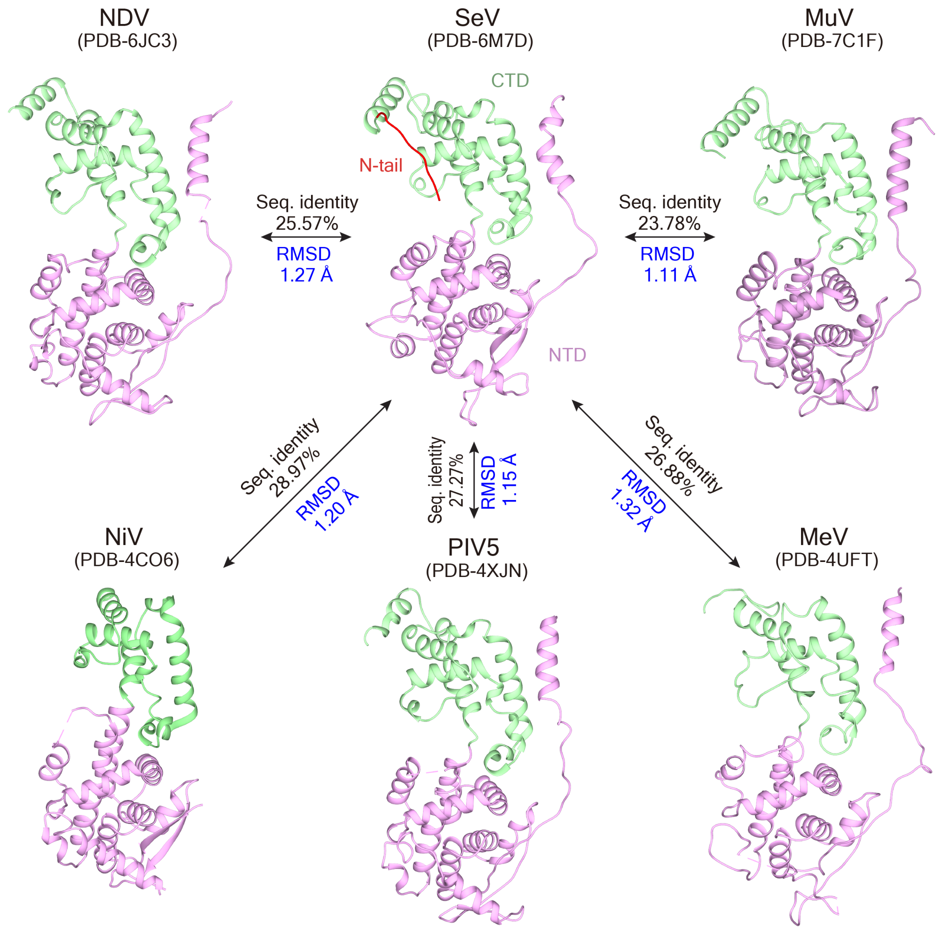

Supplementary Fig. 5 Sequence and structural comparisons of paramyxovirus nucleoproteins. Sequence identity between SeV nucleoprotein and others is marked in black. RMSD of pruned atomic pairs between SeV nucleoprotein and other paramyxovirus nucleoproteins is labeled in blue. NTD and CTD in all paramyxoviruses are colored in pink and green, respectively. SeV N-tail is colored in red.

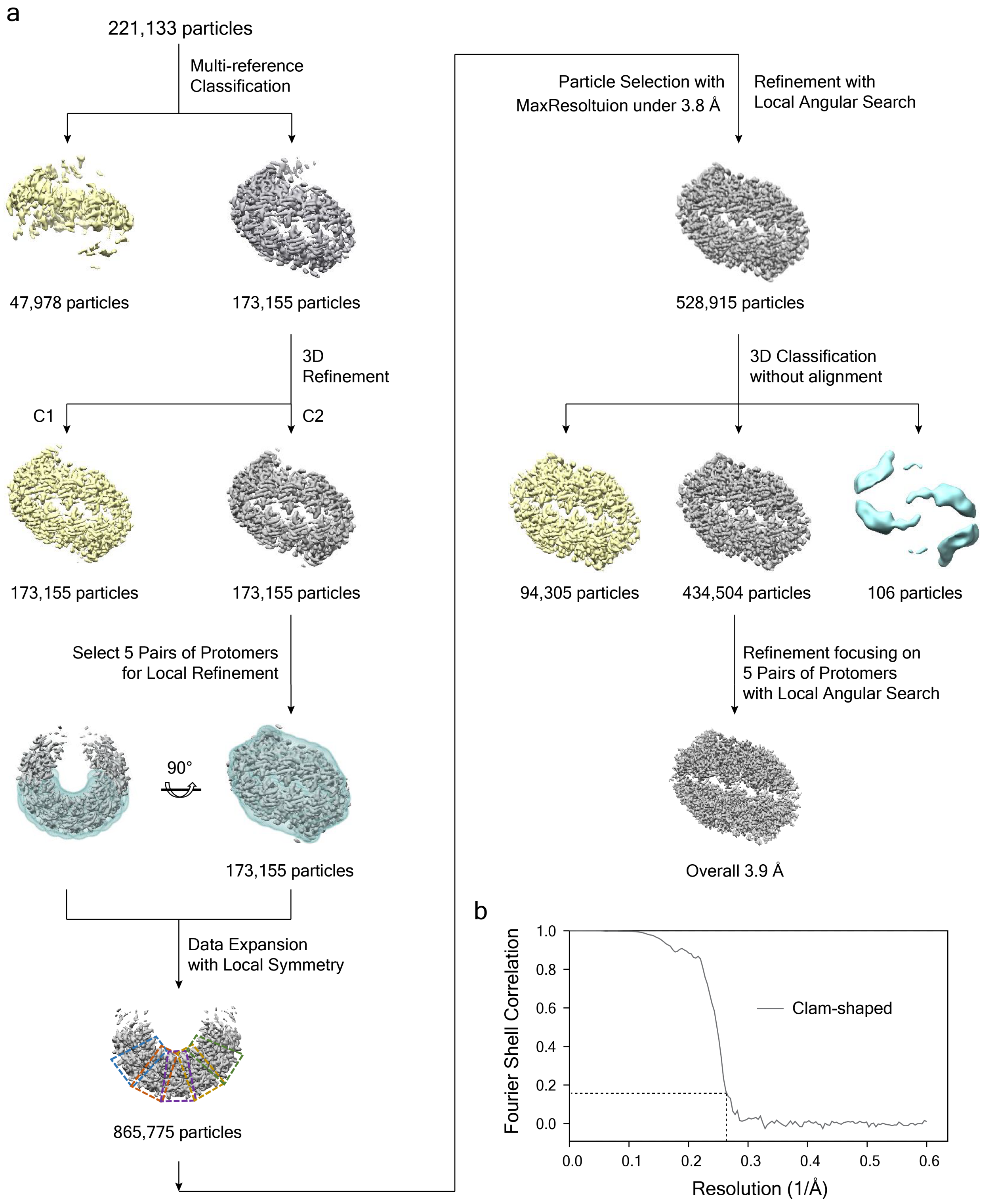

Supplementary Fig. 6 A flow chart for 3D reconstruction and local refinement of the clam-shaped structure of SeV nucleocapsid, and the FSC curve. a A flow chart for 3D reconstruction and local refinement of SeV clam-shaped structure. **b** FSC curve for SeV clam-shaped structure.

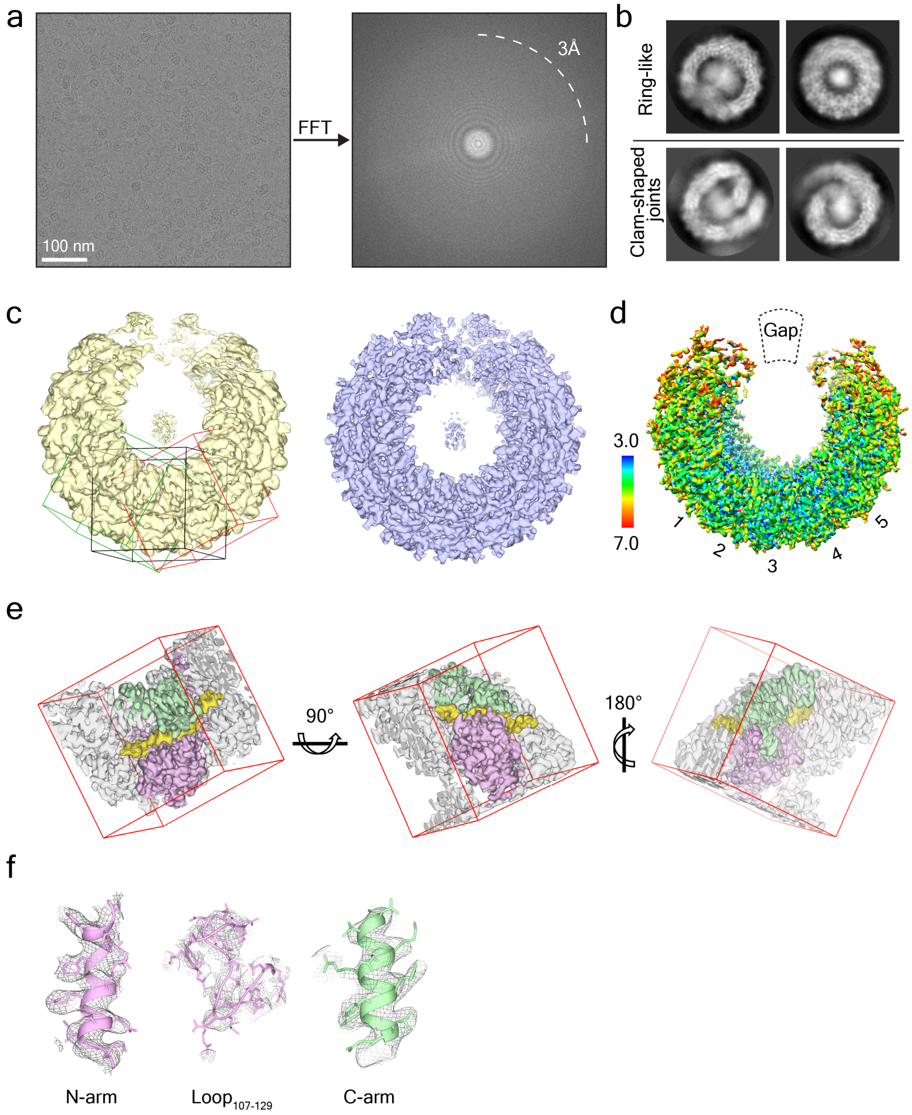

Supplementary Fig. 7 3D reconstruction of SeV clam-shaped structure. a A typical cryo-EM micrograph of SeV clam-shaped structure, and its power spectrum. **b** 2D class average galleries of SeV clam-shaped structure. The top two classes are from the dispersed ring-like particles and the bottom two are from the clam-shaped joints between double-headed nucleocapsids. **c** Two kinds of clam-shaped structures from 3D classification. Boxes in different colors in (**c**) show mask positions for local refinements. **d** Local resolution estimation of SeV clam-shaped structure via ResMap. The gap position is labeled in dashed polygon. **e** Different views of three consecutive protomers after local refinements. NTD, CTD and RNA from the middle protomer are colored in pink, green and gold, respectively. **f** Cryo-EM densities of selected areas of nucleoprotein clam-shaped structure at 3.9 Å resolution. N-arm, C-arm and $Loop_{107-129}$ are shown.

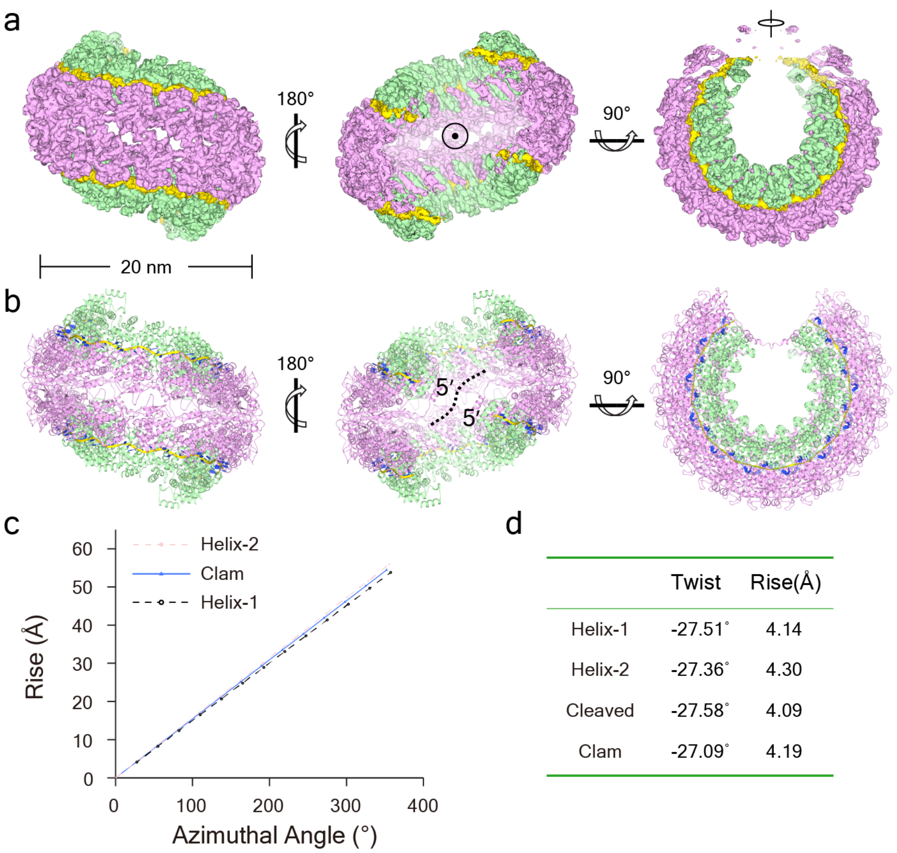

	Twist	Rise(Å)
Helix-1	-27.51˚	4.14
Helix-2	-27.36˚	4.30
Cleaved	-27.58˚	4.09
Clam	-27.09˚	4.19

Supplementary Fig. 8 3D reconstruction of SeV clam-shaped structures, and its atomic model. a 3D reconstruction of SeV clam-shaped structure. Different views are shown. The two-fold symmetrical axis enforced during reconstruction is indicated in the right two views. The NTD, CTD and RNA are colored in pink, green and gold, respectively. **b** Atomic models of SeV clam-shaped structures. Exactly the same views with (**a**) are shown. Two 5' ends of enwrapped RNA and the seam between them are labeled in the middle snapshot. **c** Plots of Z-height against the azimuthal angle among two kinds of helical stems and clam-shaped structure. **d** Summary of helical parameters of two kinds of helical stems, $NC_{cleaved}$ and clam-shaped structure.

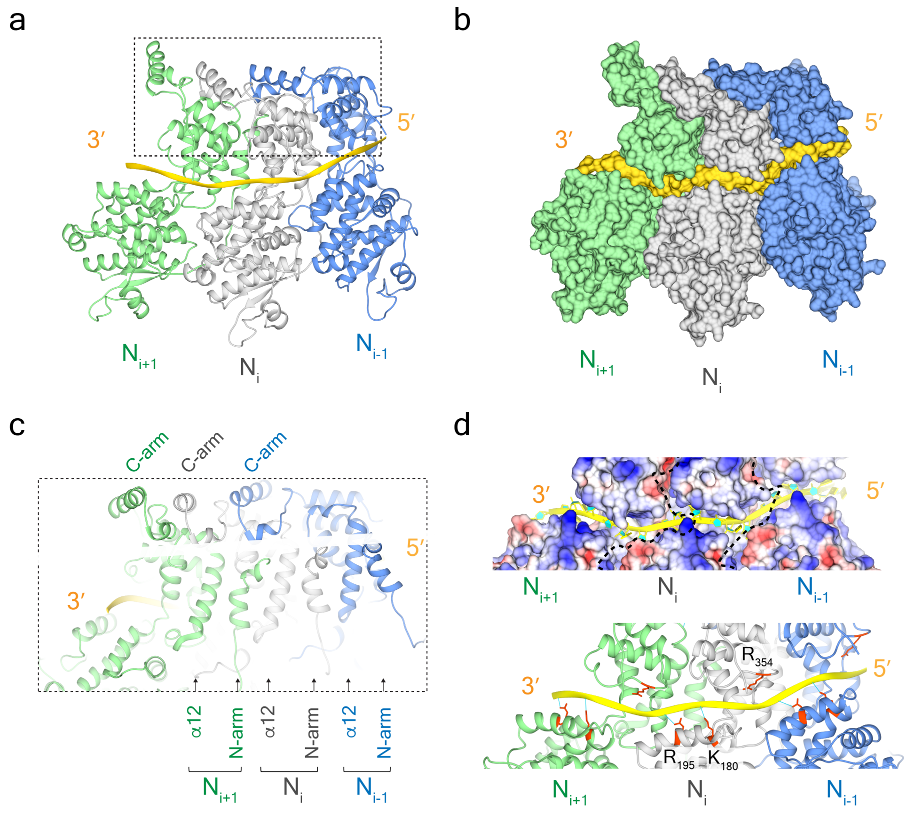

Supplementary Fig. 9 The assembly of SeV nucleocapsids via domain swapping. a The atomic model of three neighboring protomers in SeV nucleoprotein helix. Protomers are colored in blue, grey and green, respectively, and RNA is colored in gold. The view is rotated 180° against the view in Fig. 2a. The interfaces between neighboring N-arm and C-arm are boxed in dashed box. **b** Molecular surfaces of three neighboring protomers with the same view as in (**a**). **c** Domain swapping involved by C-arm (Upper) and N-arm (Lower) in three neighboring protomers. The view is similar to the dotted box in (**a**). **d** Nucleoproteins enwrap RNA via electrostatic interaction. Contour surface of electrostatic potential (Upper) and atomic model (Lower) of three neighboring nucleoproteins are shown. The borders between neighboring protomers are depicted in dotted lines in the Upper image. RNA direction is labeled in all panels.

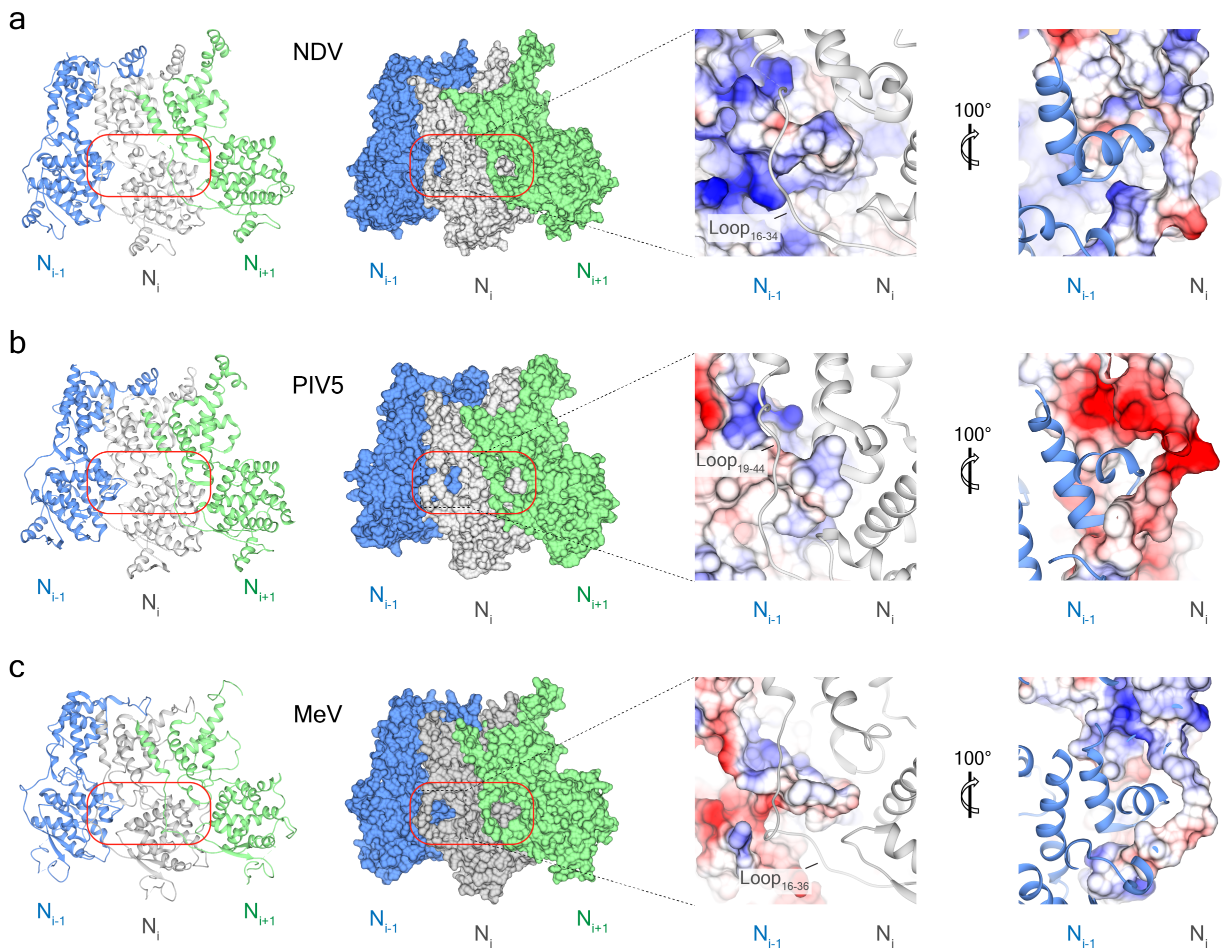

Supplementary Fig. 10 The swapped interface between the extended loops and N-holes of nucleoproteins in NDV, PIV5 and MeV. a, **b**, **c** The interface analyses on NDV, PIV5 and MeV, respectively. On the left two images, the atomic model of three neighboring protomers and their molecular surface are shown. Protomers are colored in blue, grey and green, respectively. An unnoticed swapped interface between neighboring protomers are boxed in red. On the right two images, electrostatic distribution of the extended loop and N-hole are depicted.

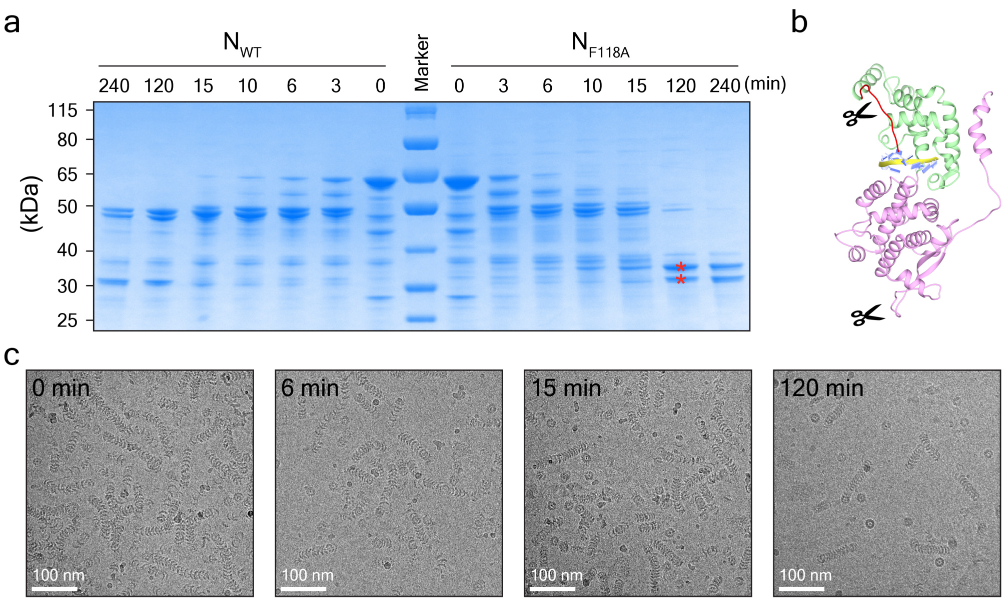

Supplementary Fig. 11 Cleavage of N-tail from SeV nucleocapsids yields straight filaments. **a** Trypsin cleavage analysis on N_{WT} (~60% double-headed nucleocapsid) and N_{F118A} (only single-headed nucleocapsid). Two extra bands (~35 kDa) are marked with red stars, after 120 min incubation. **b** Two cutting sites on nucleoproteins. **c** Typical cryo-EM micrographs of trypsin cleaved N_{WT} at indicated time points.

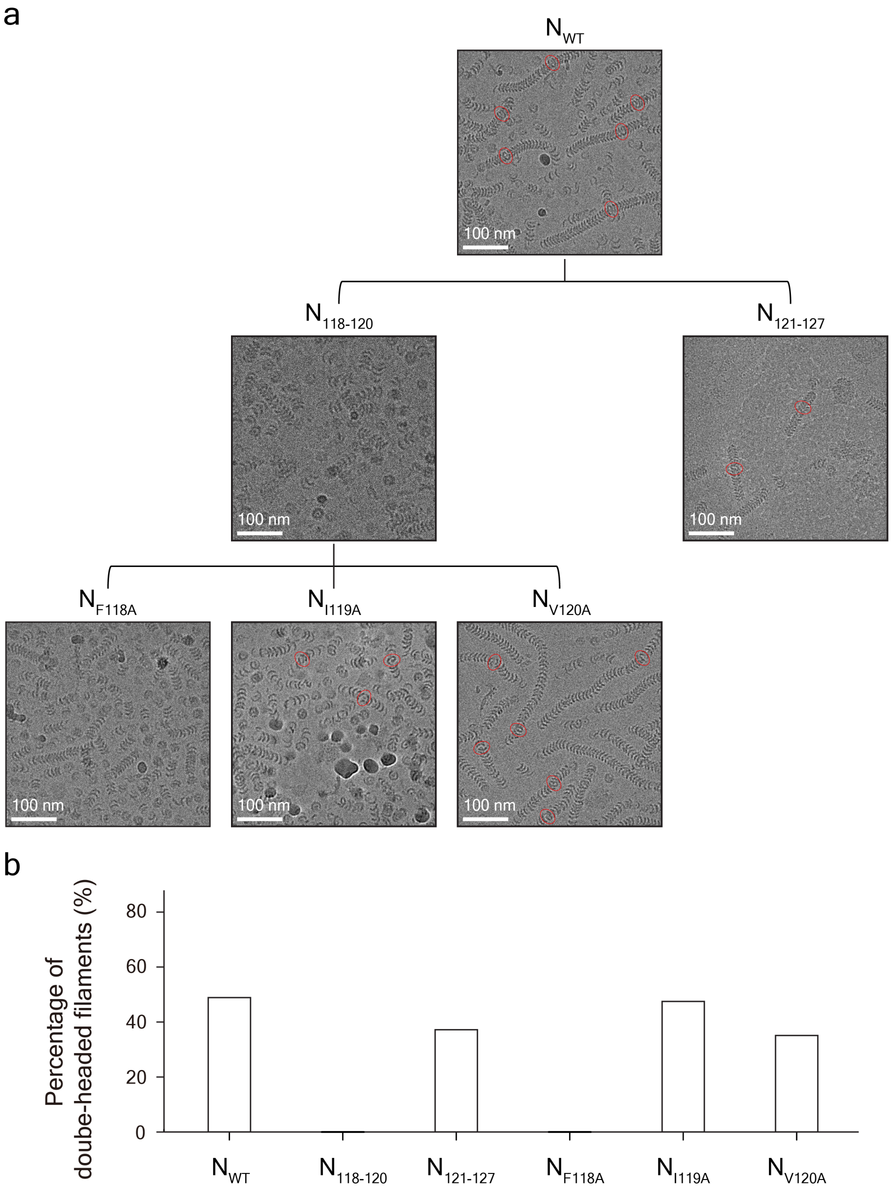

Supplementary Fig. 12 Mutant screening to abolish the formation of double-headed SeV nucleocapsid. a Typical cryo-EM images of SeV nucleoprotein mutants, and the screening pipeline. Clam-shaped structures in double-headed SeV nucleocapsids are labeled in red circles. In $N_{118\text{-}120}$, residues F118-I-V120 were replaced by AAA; in $N_{121\text{-}127}$, residues K121-TRDME-Y127 were replaced by 7 consecutive Alanine. **b** The percentage of double-headed nucleocapsids to all filaments in different SeV nucleoprotein mutants. 10 cryo-EM micrographs for each SeV nucleoprotein mutant were randomly selected and counted.

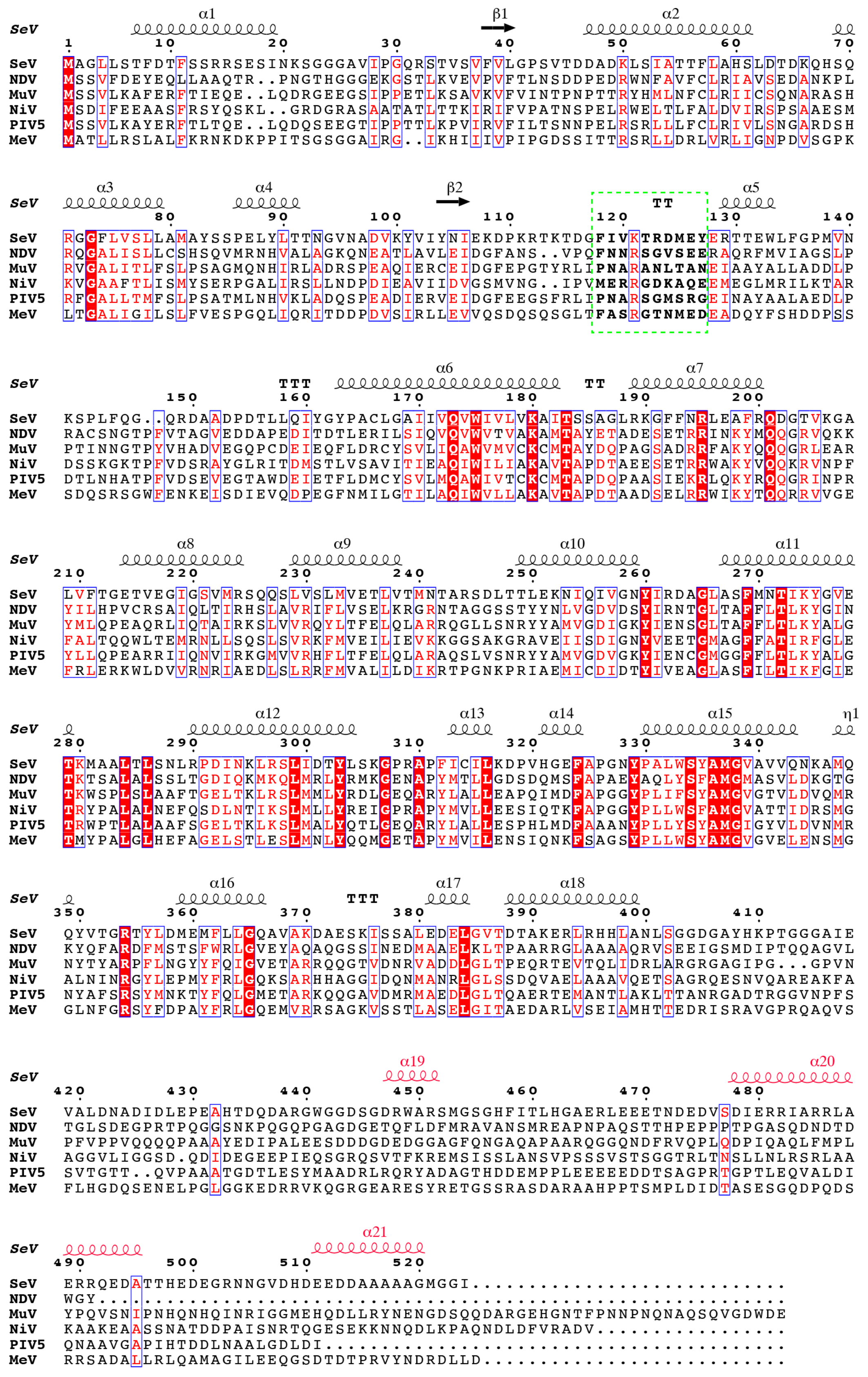
α1 β1 α2
SeV MAGLLSTFDTFSSRRSESINKSGGGAVIPGQRSTVSVFVLGPSVTDDADKLSIATTFLAHSLDTDKQHSQ
NDV MSSVFDEYEQLLAAQTR..PNGTHGGGEKGSTLKVEVPVFTLNSDDPEDRWNFAVFCLRIAVSEDANKPL
MuV MSSVLKAFERFTIEQE..LQDRGEEGSIPPETLKSAVKVFVINTPNPTTRYHMLNFCLRIICSQNARASH
NiV MSDIFEEAASFRSYQSKL..GRDGRASAATATLTTKIRIFVPATNSPELRWELTLFALDVIRSPSAAESM
PIV5 MSSVLKAYERFTLTQE..LQDQSEEGTIPPTTLKPVIRVFILTSNNPELRSRLLLFCLRIVLSNGARDSH
MeV MATLLRSLALFKRNKDKPPITSGSGGAIRG..IKHIIIVPIPGDSSITTRSRLLDRLVRLIGNPDVSGPK
α3 α4 β2 TT α5
SeV RGGFLVSLLAMAYSSPELYLTTNGVNADVKYVIYNIEKDPKRTKTDGFIVKTRDMEYERTTEWLFGPMVN
NDV RQGALISLLCSHSQVMRNHVALAGKQNEATLAVLEIDGFANS..VPQFNNRSGVSEERAQRFMVIAGSLP
MuV RVGALITLFSLPSAGMQNHIRLADRSPEAQIERCEIDGFEPGTYRLIPNARANLTANEIAAYALLADDLP
NiV KVGAAFTLISMYSERPGALIRSLLNDPDIEAVIIDVGSMVNG..IPVMERRGDKAQEEMEGLMRILKTAR
PIV5 RFGALLTMFSLPSATMLNHVKLADQSPEADIERVEIDGFEEGSFRLIPNARSGMSRGEINAYAALAEDLP
MeV LTGALIGILSLFVESPGQLIQRITDDPDVSIRLLEVVQSDQSQSGLTFASRGTNMEDEADQYFSHDDPSS
TTT α6 TT α7
SeV KSPLFQG..QRDAADPDTLLQIYGYPACLGAIIVQVWIVLVKAITSSAGLRKGFFNRLEAFRQDGTVKGA
NDV RACSNGTPFVTAGVEDDAPEDITDTLERILSIQVQVWVTVAKAMTAYETADESETRRINKYMQQGRVQKK
MuV PTINNGTPYVHADVEGQPCDEIEQFLDRCYSVLIQAWVMVCKCMTAYDQPAGSADRRFAKYQQQGRLEAR
NiV DSSKGKTPFVDSRAYGLRITDMSTLVSAVITIEAQIWILIAKAVTAPDTAEESETRRWAKYVQQKRVNPF
PIV5 DTLNHATPFVDSEVEGTAWDEIETFLDMCYSVLMQAWIVTCKCMTAPDQPAASIEKRLQKYRQQGRINPR
MeV SDQSRSGWFENKEISDIEVQDPEGFNMILGTILAQIWVLLAKAVTAPDTAADSELRRWIKYTQQRRVVGE
α8 α9 α10 α11
SeV LVFTGETVEGIGSVMRSQQSLVSLMVETLVTMNTARSDLTTLEKNIQIVGNYIRDAGLASFMNTIKYGVE
NDV YILHPVCRSAIQLTIRHSLAVRIFLVSELKRGRNTAGGSSTYYNLVGDVDSYIRNTGLTAFFLTLKYGIN
MuV YMLQPEAQRLIQTAIRKSLVVRQYLTFELQLARRQGLLSNRYYAMVGDIGKYIENSGLTAFFLTLKYALG
NiV FALTQQWLTEMRNLLSQSLSVRKFMVEILIEVKKGGSAKGRAVEIISDIGNYVEETGMAGFFATIRFGLE
PIV5 YLLQPEARRIIQNVIRKGMVVRHFLTFELQLARAQSLVSNRYYAMVGDVGKYIENCGMGGFFLTLKYALG
MeV FRLERKWLDVVRNRIAEDLSLRRFMVALILDIKRTPGNKPRIAEMICDIDTYIVEAGLASFILTIKFGIE
α12 α13 α14 α15 η1
SeV TKMAALTLSNLRPDINKLRSLIDTYLSKGPRAPFICILKDPVHGEFAPGNYPALWSYAMGVAVVQNKAMQ
NDV TKTSALALSSLTGDIQKMKQLMRLYRMKGENAPYMTLLGDSDQMSFAPAEYAQLYSFAMGMASVLDKGTG
MuV TKWSPLSLAAFTGELTKLRSLMMLYRDLGEQARYLALLEAPQIMDFAPGGYPLIFSYAMGVGTVLDVQMR
NiV TRYPALALNEFQSDLNTIKSLMLLYREIGPRAPYMVLLEESIQTKFAPGGYPLLWSFAMGVATTIDRSMG
PIV5 TRWPTLALAAFSGELTKLKSLMALYQTLGEQARYLALLESPHLMDFAAANYPLLYSYAMGIGYVLDVNMR
MeV TMYPALGLHEFAGELSTLESLMNLYQQMGETAPYMVILENSIQNKFSAGSYPLLWSYAMGVGVELENSMG
α16 TTT α17 α18
SeV QYVTGRTYLDMEMFLLGQAVAKDAESKISSALEDELGVTDTAKERLRHHLANLSGGDGAYHKPTGGGAIE
NDV KYQFARDFMSTSFWRLGVEYAQAQGSSINEDMAAELKLTPAARRGLAAAAQRVSEEIGSMDIPTQQAGVL
MuV NYTYARPFLNGYYFQIGVETARRQQGTVDNRVADDLGLTPEQRTEVTQLIDRLARGRGAGIPG...GPVN
NiV ALNINRGYLEPMYFRLGQKSARHHAGGIDQNMANRLGLSSDQVAELAAAVQETSAGRQESNVQAREAKFA
PIV5 NYAFSRSYMNKTYFQLGMETARKQQGAVDMRMAEDLGLTQAERTEMANTLAKLTTANRGADTRGGVNPFS
MeV GLNFGRSYFDPAYFRLGQEMVRRSAGKVSSTLASELGITAEDARLVSEIAMHTTEDRISRAVGPRQAQVS
α19 α20
SeV VALDNADIDLEPEAHTDQDARGWGGDSGDRWARSMGSGHFITLHGAERLEEETNDEDVSDIERRIARRLA
NDV TGLSDEGPRTPQGGSNKPQGQPGAGDGETQFLDFMRAVANSMREAPNPAQSTTHPEPPPTPGASQDNDTD
MuV PFVPPVQQQQPAAAYEDIPALEESDDDGDEDGGAGFQNGAQAPAARQGGQNDFRVQPLQDPIQAQLFMPL
NiV AGGVLIGGSD.QDIDEGEEPIEQSGRQSVTFKREMSISSLANSVPSSSVSTSGGTRLTNSLLNLRSRLAA
PIV5 SVTGTT..QVPAAATGDTLESYMAADRLRQRYADAGTHDDEMPPLEEEEEDDTSAGPRTGPTLEQVALDI
MeV FLHGDQSENELPGLGGKEDRRVKQGRGEARESYRETGSSRASDARAAHPPTSMPLDIDTASESGQDPQDS
α21
SeV ERRQEDATTHEDEGRNNGVDHDEEDDAAAAAGMGGI.............................
NDV WGY..
MuV YPQVSNIPNHQNHQINRIGGMEHQDLLRYNENGDSQQDARGEHGNTFPNNPNQNAQSQVGDWDE
NiV KAAKEAASSNATDDPAISNRTQGESEKKNNQDLKPAQNDLDFVRADV.................
PIV5 QNAAVGAPIHTDDLNAALGDLDI...
MeV RRSADALLRLQAMAGILEEQGSDTDTPRVYNDRDLLD............................

Supplementary Fig. 13 Sequences alignment of nucleoproteins from different species in the family of *Paramyxoviridae*. α1-α18 helices from the atomic model are labeled on the respective sequences. The predicted α19-α21 helices on the unstructured N-tail are labeled in red.

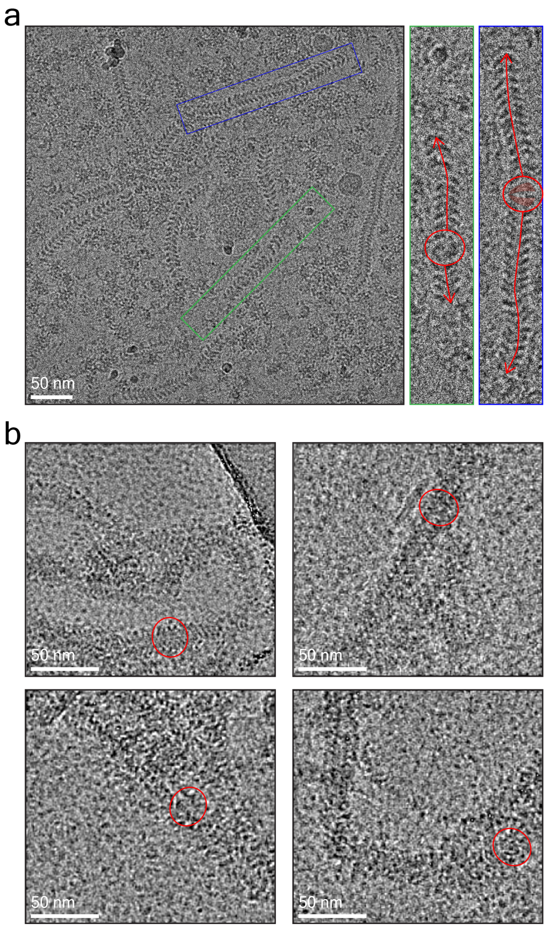

Supplementary Fig. 14 Double-headed SeV nucleocapsids overexpressed in HEK293F cells and isolated from SeV virion. a A typical cryo-EM micrograph of double-headed SeV nucleocapsids overexpressed in HEK293F cells. Two double-headed filaments are boxed in green and blue, and zoomed in. The trajectory and the clam-shaped joint in each filament are depicted in red. **b** Image gallery of double-headed SeV nucleocapsids isolated from virion. Sharpening is applied to the whole micrograph and typical clam-shaped joint in each micrograph is circled in red.
