# Supplementary material for: Structure and assembly of double-headed Sendai virus nucleocapsids
Source: Commun Biol. 2021 Apr 22;4:494. doi: 10.1038/s42003-021-02027-y (PMC8062630; doi:10.1038/s42003-021-02027-y)
Supplement: Supplementary file 4 — Descriptions of Additional Supplementary Files [file 42003_2021_2027_MOESM4_ESM.pdf]

Descriptions of additional supplementary files

Supplementary Movie 1
Description: 3D reconstruction of SeV NCcleaved and the fitting of atomic model.

Supplementary Movie 2
Description: 3D reconstruction of SeV clam-shaped structure and the fitting of atomic model.

Supplementary Movie 3
Description: Assembly mechanism of SeV nucleocapsid. Domain swapping processes involved by N-arm, C-arm and the unnoticed N-hole are displayed sequentially.

Supplementary Movie 4
Description: Interface analysis between opposite rungs in SeV clam-shaped structure.

Supplementary Movie 5
Description: Morphing from closed NDV clam-shaped structure to hyperclosed SeV clam-shaped structure. The initial frame is the NDV clam-shaped structure and the last frame is the SeV clam-shaped structure.
